# [Ag_9_(1,2-BDT)_6_]^3–^: How Square-Pyramidal Building Blocks Self-Assemble into the Smallest Silver Nanocluster

**DOI:** 10.1021/acs.inorgchem.1c00334

**Published:** 2021-03-17

**Authors:** Badriah J. Alamer, Megalamane S. Bootharaju, Sergey M. Kozlov, Zhen Cao, Aleksander Shkurenko, Saidkhodzha Nematulloev, Partha Maity, Omar F. Mohammed, Mohamed Eddaoudi, Luigi Cavallo, Jean-Marie Basset, Osman M. Bakr

**Affiliations:** ⊥ Division of Physical Sciences and Engineering, King Abdullah University of Science and Technology (KAUST), Thuwal 23955-6900, Saudi Arabia; † KAUST Catalysis Center, Division of Physical Sciences and Engineering, King Abdullah University of Science and Technology (KAUST), Thuwal 23955-6900, Saudi Arabia; ° Department of Chemistry, College of Sciences, Taif University, Taif 11099, Saudi Arabia; ‡ Center for Nanoparticle Research, Institute for Basic Science, Seoul 08826, Republic of Korea; ∇ School of Chemical and Biological Engineering and Institute of Chemical ProcessesSeoul National University, Seoul 08826, Republic of Korea; § Department of Chemical and Biomolecular Engineering, Faculty of Engineering, National University of Singapore, Singapore 119260, Singapore; ∧ Functional Materials Design, Discovery and Development Research Group, Advanced Membranes and Porous Materials Center, King Abdullah University of Science and Technology (KAUST), Thuwal 23955-6900, Saudi Arabia; ¶ Advanced Membranes and Porous Materials Center, Division of Physical Sciences and Engineering, King Abdullah University of Science and Technology (KAUST), Thuwal 23955-6900, Saudi Arabia

## Abstract

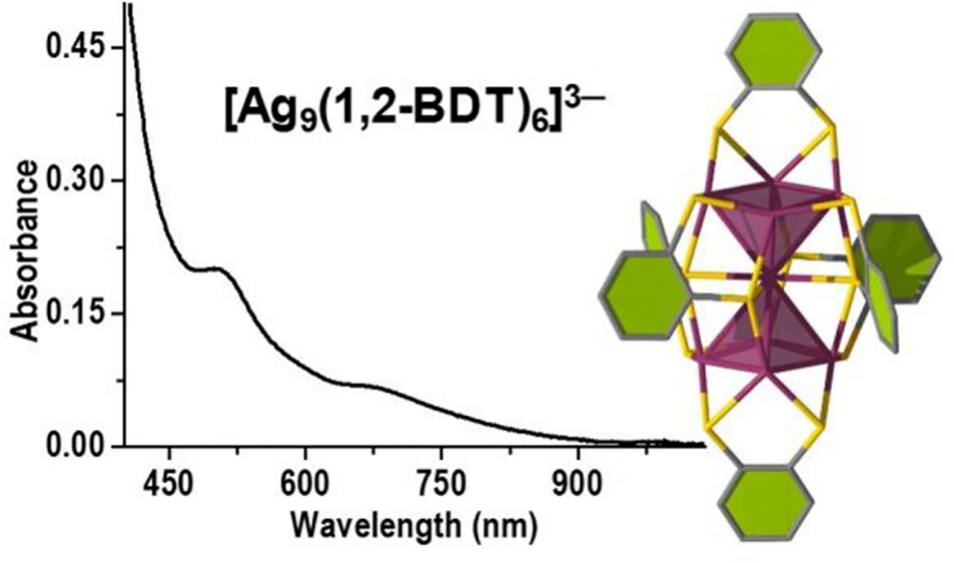

The emerging promise of few-atom metal catalysts has driven the need for developing metal nanoclusters (NCs) with ultrasmall core size. However, the preparation of metal NCs with single-digit metallic atoms and atomic precision is a major challenge for materials chemists, particularly for Ag, where the structure of such NCs remains unknown. In this study, we developed a shape-controlled synthesis strategy based on an isomeric dithiol ligand to yield the smallest crystallized Ag NC to date: [Ag_9_(1,2-BDT)_6_]^3–^ (1,2-BDT = 1,2-benzenedithiolate). The NC’s crystal structure reveals the self-assembly of two Ag square pyramids through preferential pyramidal vertex sharing of a single metallic Ag atom, while all other Ag atoms are incorporated in a motif with thiolate ligands, resulting in an elongated body-centered Ag_9_ skeleton. Steric hindrance and arrangement of the dithiolated ligands on the surface favor the formation of an anisotropic shape. Time-dependent density functional theory based calculations reproduce the experimental optical absorption features and identify the molecular orbitals responsible for the electronic transitions. Our findings will open new avenues for the design of novel single-digit metal NCs with directional self-assembled building blocks.

## Abstract 2

An isomeric dithiol-based synthesis strategy is used to synthesize the smallest crystallized Ag nanocluster, [Ag_9_(1,2-BDT)_6_]^3−^. The single-digit cluster reveals novel bonding modes and building blocks, which include the self-assembly of two Ag square pyramids sharing a common vertex and six bidentate thiolate motifs.

## Introduction

Investigation of the synthesis and chemistry of noble-metal nanoclusters (NCs), such as Au and Ag, is extensively growing^1−4^ and has set the scene for establishing these materials as promising candidates for an expansive range of applications, including medical therapy,^5^ drug delivery,^6^ sensing,^7,8^ and catalysis.^9−11^ NCs are particularly desirable for catalysis because of their high catalytic activity and/or selectivity.^12,13^ The major conceptual driver for NC utilization in catalysis is based on maximization of the per-atom reaction efficiency through control of the metal particle’s size from the nanometer to subnanometer scale and ultimately to a few single atoms.^14,15^ Therefore, NCs exhibit a range of unique and often unexpected properties compared to larger nanoparticles and bulk materials. This distinct behavior is due to a variety of factors, including the quantum size effect, geometric shell closing,^16^ and low-coordination environment. Recently, considerable efforts were devoted to the synthesis of atomically precise clusters with well-defined, stable, and tunable compositions,^17,18^ with the aim of achieving a rigorous basis for understanding the correlation between the NCs’ structures and their properties.

Metal NCs with single-digit metallic atoms present the highest potential for catalytic activity and also the closest bridge to atomic-level behavior. Metal NCs with fewer than 10 metal atoms not only are good candidates for catalysis but also serve as ideal models to explore the relationship between the metal interface, protecting ligands, and their catalytic activities.^15^ Unfortunately, the fabrication of stable thiolated NCs of Ag, Au, or Cu, with less than 10 atoms, remains difficult to achieve compared to the easier preparation of larger particles.

To date, most of the well-known examples of monolayer-protected Ag NCs are large and quasi-spherical,^19−25^ whereas only a few limited NCs displayed anisotropic geometries.^26−29^ However, there are studies regarding smaller species (*n* < 10) that differ in the size and composition with various types of capping ligands, namely, Ag_7_(DMSA)_4_,^30^ Ag_8_(H_2_MSA)_8_,^31^ and Ag_9_(H_2_MSA)_7_.^32^ These limited numbers of species—nominally assigned as NCs based on their compositions and molecular weights have unknown crystal structures. Furthermore, stability remains the main obstacle in the synthesis and crystallization of Ag NCs with fewer than 10 metal atoms because Ag is prone to oxidation and is sensitive to light. In Au, while there are several phosphine-protected NCs with few metal atoms,^33,34^ the relatively large thiolate Au cluster Au_15_(SR)_13_^35^ is considered to be the smallest NC so far. Only in Cu has the synthesis and crystallization of clusters with fewer than 10 metal atoms been achieved in the form of Cu_6_(SR)_6_. However, it can be easily oxidized upon exposure to air.^36^ These observations inspired us to investigate chemical approaches in order to synthesize stable small silver NCs because no sizes between the [Ag^I^(SR)] complexes and [Ag_*n*_(SR)_*m*_] (10 > *n* > *m*) have been crystallized and structurally solved. In this work, we employed the 1,2-benzenedithiolate (1,2-BDT) ligand to synthesize a Ag NC, [Ag_9_(1,2-BDT)_6_]^3–^. The crystal structure comprises a Ag_9_ metal skeleton formed by the self-assembly of two Ag square pyramids through preferential pyramidal vertex sharing. The optical properties are studied in detail using density functional theory (DFT) calculations.

## Results and Discussion

In the majority of NC syntheses, the size, structure, and properties are controlled by the protecting ligands.^37−40^ We reasoned that using a small-footprint bidentate thiol such as 1,2-BDT plays an important role in controlling the size of the NC. The steric hindrance between the two adjacent thiolate groups via S lone-pair electron repulsions and the short distance between S binding sites could terminate the growth of a smaller size cluster. In addition to the ligand structure, other reaction conditions, such as the reducing agent and temperature, are also known to influence the NC size.^41^ Considering these facts, our synthesis of [Ag_9_(1,2-BDT)_6_](TOA)_3_ NCs (Figure S1) involves the chemical reduction of silver thiolates [Ag-(1,2-BDT)] in mixed solvents of methanol and dichloromethane (DCM) by an aqueous sodium borohydride (NaBH_4_) solution in the presence of tetraoctylammonium (TOA) countercations (see the Experimental Section for details). After the reaction, the synthesized product was washed by a solvent to remove excess reagents.

The final purified product dissolved in DCM shows well-defined peaks at 667 and 504 nm in its UV–vis absorption spectrum (Figure S2), which is completely different from that of [Ag_29_(1,3-BDT)_12_]^3–^ NC,^42^ whose prominent absorption peak appears at 440 nm. The absence of the surface-plasmon-resonance peak for Ag nanoparticles and the presence of multiple peaks suggest the formation of a new cluster size with 1,2-BDT. The purified NC product in a dimethylformamide solution was layered with ethanol (see the Experimental Section for details) to obtain single crystals in order to determine the chemical formula, electronic charge, and molecular structure of the NC.

**Figure 1:**
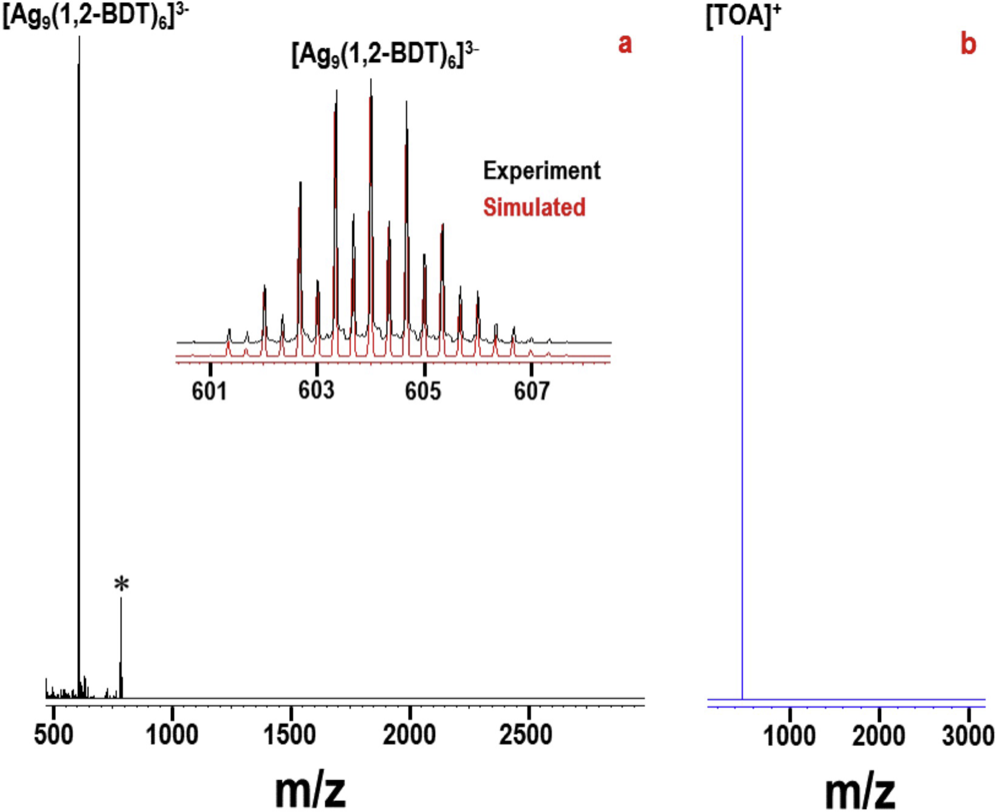
ESI-MS of [Ag_9_(1,2-BDT)_6_]^3–^ NCs in (a) negative-ion and (b) positive-ion modes. Inset of part a: Comparison of a simulated mass spectrum for [Ag_9_(1,2-BDT)_6_]^3–^ with the experiment. The asterisk peak in part a is due to the fragment [Ag_8_(1,2-BDT)_5_]^2–^, originating from its parent cluster [Ag_9_(1,2-BDT)_6_]^3–^.

Electrospray ionization mass spectrometry (ESI-MS) of a single-crystal solution of the NCs showed a prominent peak at *m*/*z* 604 in negative-ion mode (Figure 1a). Expansion of this peak revealed a characteristic Ag isotopic pattern with a peak separation of *m*/*z* 0.33 (inset of Figure 1a), suggesting the charge of the molecular ion to be 3–. Considering Ag and BDT, the total mass of this ion for the *m*/*z* 604 peak (i.e., 604 × 3 = 1812) was assigned to a composition of [Ag_9_(1,2-BDT)_6_]^3–^. This assigned composition was further confirmed by comparing a simulated mass spectrum for [Ag_9_(1,2-BDT)_6_]^3–^ with that of the experiment, wherein they matched perfectly (inset of Figure 1a). Along with the prominent [Ag_9_(1,2-BDT)_6_]^3–^ peak, a minor peak for [Ag_8_(1,2-BDT)_5_]^2–^ was also observed (denoted with an asterisk in Figure 1a), originating from [Ag_9_(1,2-BDT)_6_]^3–^ by the loss of one [Ag(1,2-BDT)] unit, which is a common occurrence in ESI-MS of Ag NCs.^43^ The positive-ion-mode ESI-MS (Figure 1b) showed a single peak for [TOA]^+^, indicating stabilization of the [Ag_9_(1,2-BDT)_6_]^3–^ clusters with counterions of [TOA]^+^.

Analysis of the collected single-crystal X-ray diffraction data further validated the overall composition of the NCs that was deduced from ESI-MS. The [Ag_9_(1,2-BDT)_6_]^3–^ NCs crystallized in the monoclinic space group *P*2_1_/*c* (Table S1). Its unit cell and the packing of NCs are shown in Figure S3, revealing three TOA^+^ ions per cluster, confirming the 3– charge state of the Ag_9_ cluster.

**Figure 2:**
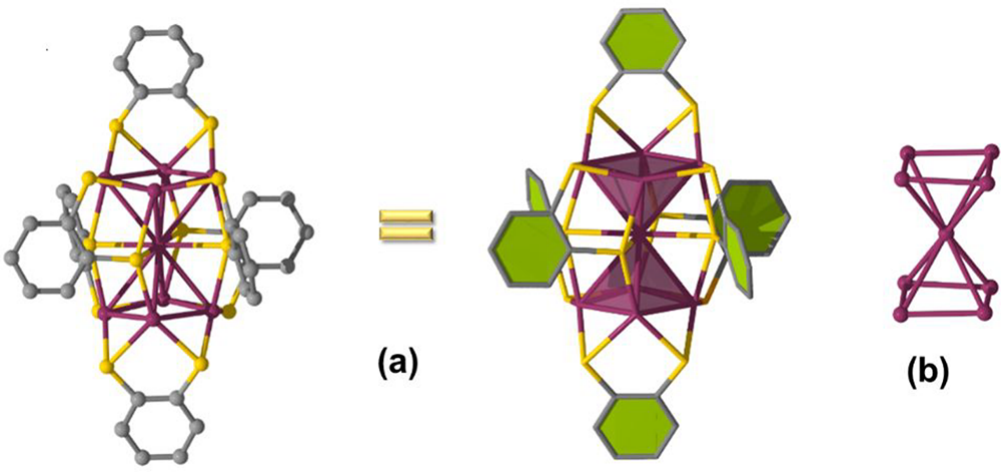
Crystal structure of the Ag_9_ NC: (a) whole [Ag_9_(1,2-BDT)_6_]^3–^ anion; (b) Ag_9_ metal skeleton of the [Ag_9_(1,2-BDT)_6_]^3–^ cluster. Color legends: gray, C; plum, Ag; yellow, S.

**Figure 3:**
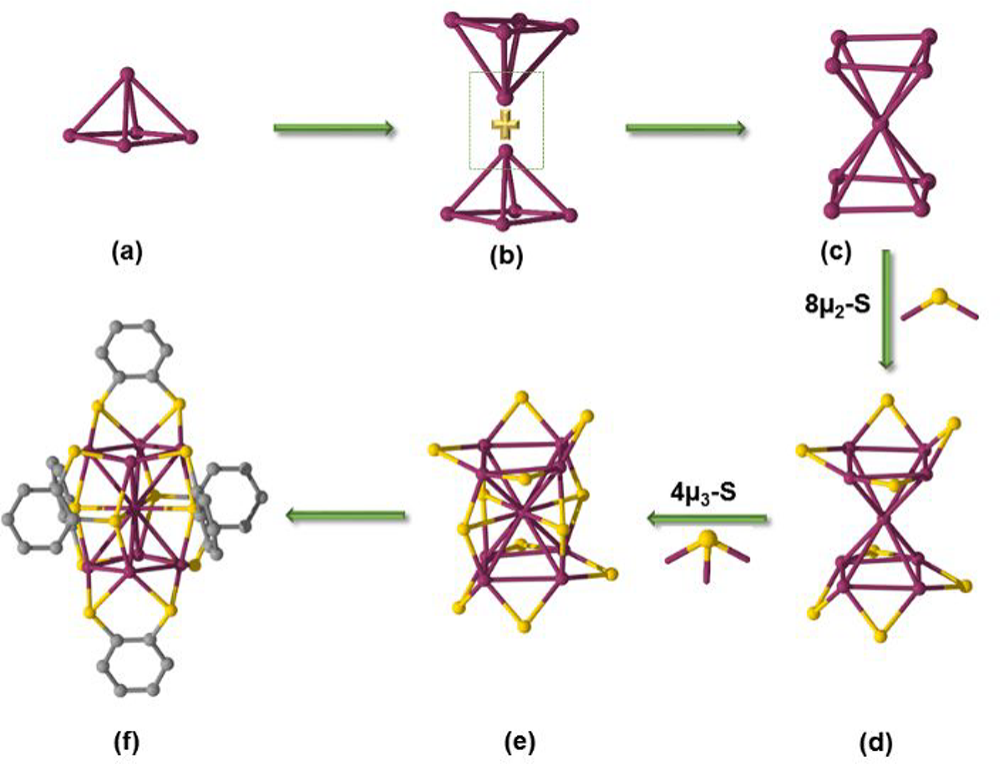
Construction of the [Ag_9_(1,2-BDT)_6_]^3–^ NC. (a) Ag_5_ of the square-pyramidal building block. (b) Linear assembly of two square pyramids through pyramidal vertex sharing to form the Ag_9_ skeleton structure as shown in part c. (d) Formation of the Ag_9_S_8_ framework upon bridging of the eight edges with eight μ_2_-S atoms of the 1,2-BDT ligands. (e) Bridging of Ag_9_S_8_ with four μ_3_-S atoms of the 1,2-BDT ligands to form a rod-shaped Ag_9_S_12_ unit. The C atoms of ligands are omitted in parts d and e for clarity. (f) Total structure of the NC. Color legends: gray, C; plum, Ag; yellow, S.

The structure of a single nonanuclear Ag cluster is shown in Figure 2a. The asymmetric unit contains the halves of the Ag nonanuclear clusters. Both halves adopt the shape of two deformed square pyramids sharing one Ag atom. Hence, the shared Ag atom appears at the center of the cluster surrounded by eight Ag corner atoms (Figure 2b). Distances between the central Ag atom and each of the remaining eight are in the ranges of 3.096(1)–3.292(1) and 3.0322(9)–3.3232(9) Å in the cases of clusters I and II, respectively (Figure S4). Therefore, the Ag–Ag distances in the [Ag_9_(BDT)_6_]^3–^ anions are definitely longer than the Ag–Ag distance of 2.88 Å in bulk Ag, indicating weak interactions among the Ag atoms. Each cluster is capped by six bidentate thiolate ligands, with two in the apical positions and four in the equatorial plane coordinated in two different connectivity modes: μ_3_-η^1^:η^2^:η^1^ and μ_4_-η^1^:η^2^:η^1^:η^1^, respectively. There are eight μ_2_-S atoms bonded to the square base of each pyramid to form Ag_9_S_8_ and four μ_3_-S atoms shared in the center and connected to the top and bottom Ag atoms of the Ag_9_ core to give the final framework of a rod-shaped Ag_9_(SR)_12_ cluster (Figure 3). The packing structure of the crystal clearly reveals the location of the counterions, [TOA]^+^ between the [Ag_9_(1,2-BDT)_6_]^3–^ anions, thus balancing the overall charge of the crystal, and the clusters of the same type are located at the same coordinate *x*, forming the 2D layers in the structure parallel to the plane (011) (Figure S3). An overlay of the two crystallographically independent clusters reveals their similarity (Figure S4). Thus, the root-mean-square deviation for the overlay equals 0.1444 Å, and the maximum distance between two equivalent atoms in the overlay (Max. D) is 0.2995 Å (excluding H atoms). The formation of a Ag_9_ skeleton can also be viewed as the simultaneous interaction of two Ag_4_ squares and a single Ag atom, as shown in Figure S5. Upon capping of this Ag_9_ metal unit with six 1,2-BDT ligands, the total structure of [Ag_9_(1,2-BDT)_6_]^3–^ is obtained.

The photophysical properties of [Ag_9_(1,2-BDT)_6_]^3–^ NCs were further studied using time-resolved photoluminescence (PL) and femtosecond transient absorption (fs-TA) spectroscopies (see the Supporting Information for details). This cluster shows a broad PL peak in the near-IR region with a maximum intensity at ∼820 nm (excitation = 480 nm; Figure S6A). This emission may be originating from the direct electron–hole recombination. The PL excitation spectrum (Figure S6B) for emission at 820 nm is found to be similar to the absorption spectrum, suggesting that PL is emanating from the NC’s core. Moreover, the PL emission and excitation maps indicated that this emission is arising from a single species, i.e., [Ag_9_(1,2-BDT)_6_]^3–^ (Figure S6C). Notably, the emission peak position of the larger-sized [Ag_29_(1,3-BDT)_12_]^3–^ cluster is 659 nm, which is higher in energy compared to that of [Ag_9_(1,2-BDT)_6_(TPP)_4_]^3–^ (820 nm).^42^ This suggests that PL of the NCs in this size regime is largely electronic-structure-dependent rather than being dominated by quantum size effects. It should be noted that the time-resolved PL data (Figure S7) show an average lifetime of 1.43 ns. This ultrafast excited-state relaxation is further supported by fs-TA spectroscopy, which shows two excited-state decay constants of 59 ps (42%) and 1.5 ns (58%) (Figure S8). The fast component could be attributed to the carrier recombination, which is likely assisted by surface trap states.

## Computational Modeling

Time-dependent DFT (TDDFT) was used to theoretically investigate the optical and electronic transitions in the [Ag_9_(1,2-BDT)_6_]^3–^ NC. The geometry of the NCs was optimized with the Tao, Perdew, Staroverov, and Scuseria^44,45^ exchange-correlation function augmented with Grimme D3 corrections,^46^ as implemented in the *ADF* software. Relativistic effects were treated using the zeroth-order regular approximation, and the COSMO implicit solvent model^47−49^ was employed to describe the solvent effect. Thereafter, the optical absorption properties of this cluster were calculated using TDDFT with the same setup. A Lorentzian broadening of 0.1 eV was applied to TDDFT excitation energies to generate the spectrum. There was less than a 0.1 eV difference between the peak positions in the calculated and experimental spectra.

**Figure 4:**
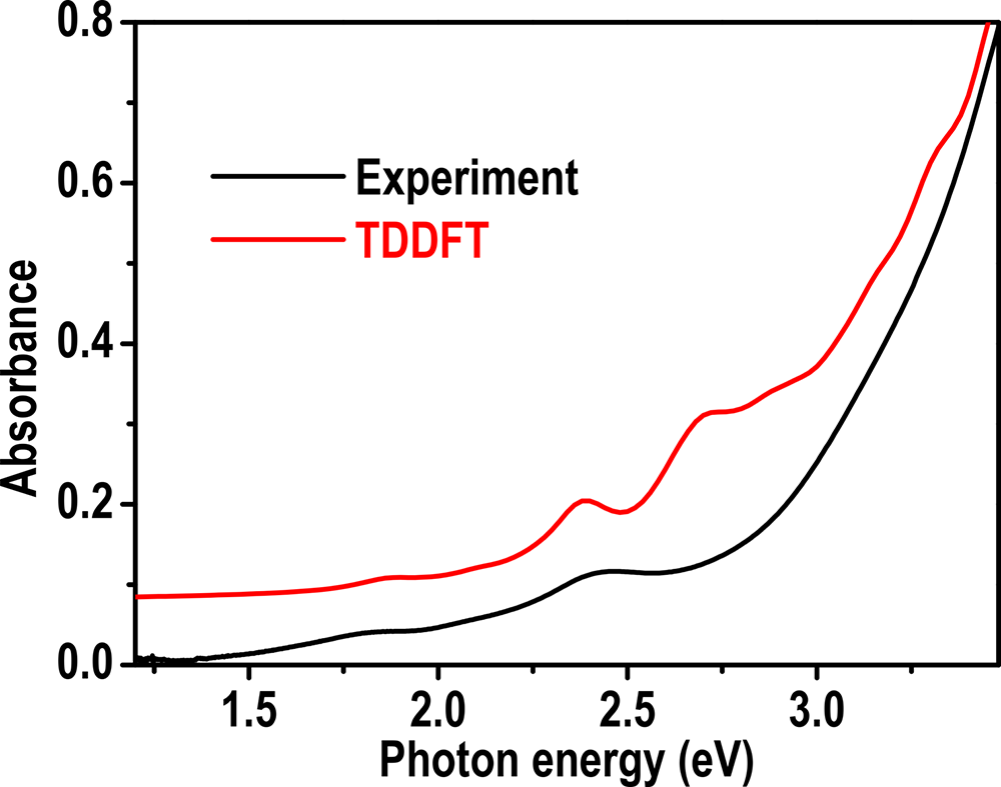
Experimental (black curve) and simulated (red curve) UV–vis absorption spectra of the [Ag_9_(1,2-BDT)_6_]^3–^ NCs.

**Figure 5:**
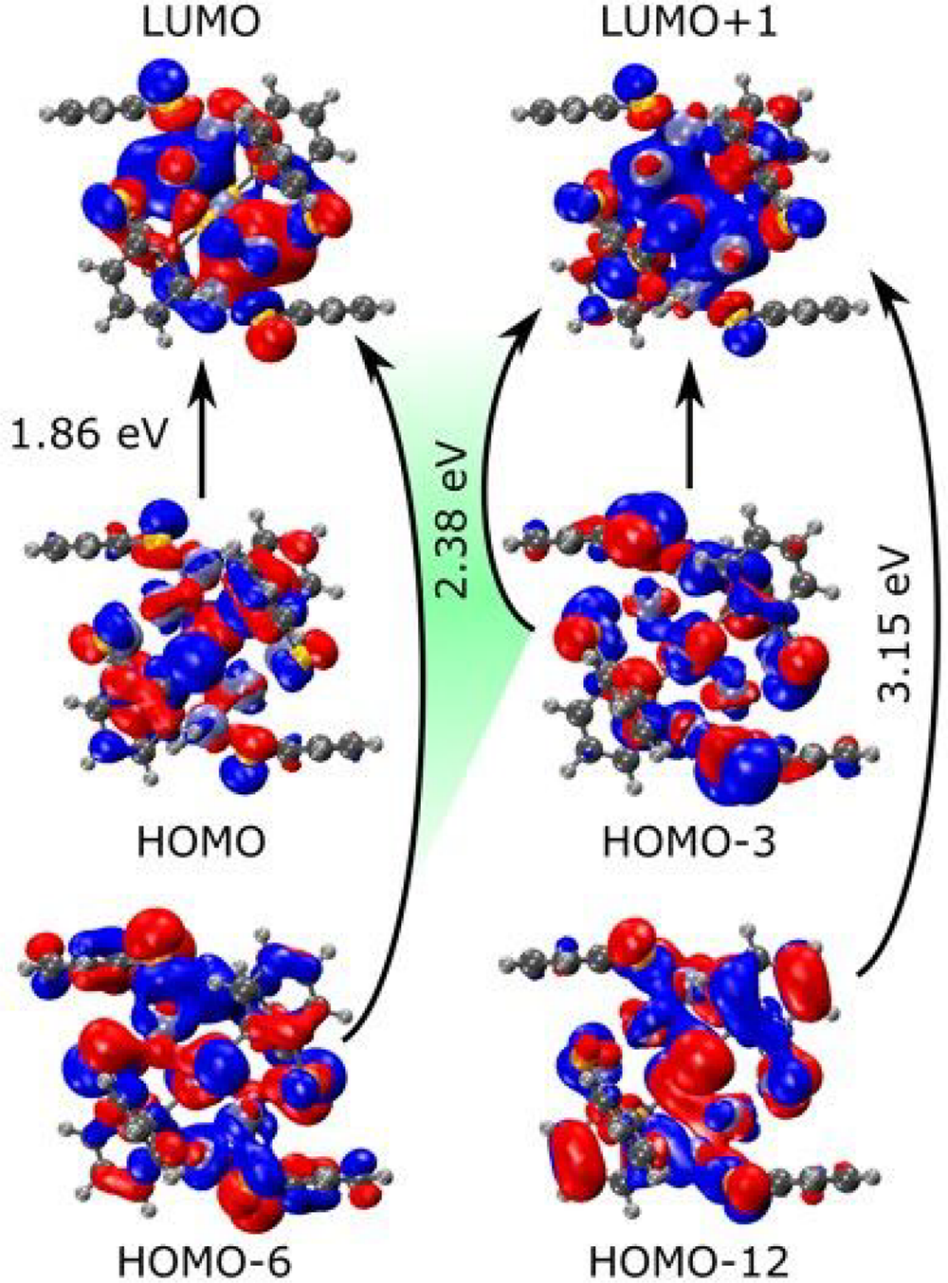
Molecular orbitals with the highest contributions to the important transitions of [Ag_9_(1,2-BDT)_6_]^3–^ NCs at ∼1.9 and ∼2.4 eV.

Our TDDFT modeling reasonably reproduced the main peaks and shoulders observed in the experimental spectrum (Figure 4). We further projected the main transition features onto the molecular orbitals (Figure 5), which show the large contribution of the ligands to the electronic structure due to the small cluster size. The highest occupied molecular orbitals (HOMOs) of the cluster have contributions from both the p system of the ligands and the d orbitals of the Ag atoms. Whereas the lowest unoccupied molecular orbital (LUMO) resembles a p-shaped superatomic orbital centered on the Ag core of the cluster, other unoccupied orbitals have more complex shapes that cannot be easily assigned to any specific type. The experimental peak at 1.86 eV (667 nm) corresponds to the calculated peak at 1.9 eV, which is essentially HOMO-to-LUMO excitation (99%). The second experimental feature at 2.46 eV (504 nm) corresponds to the simulated peak at 2.39 eV, which comes mainly from the HOMO–6-to-LUMO+1 excitation (69%). The main absorption area is a combination of several transitions, like the ones observed at 3.15 and 3.49 eV. Table S2 and Figure S9 provide more details on the orbital contributions to important optical transitions. The molar absorption coefficients of [Ag_9_(1,2-BDT)_6_](TOA)_3_ NC are found to be ∼0.4 × 10^4^ and ∼0.11 × 10^4^ M^–1^cm^–1^ for the absorption peaks at 504 and 667 nm, respectively, in DCM. In the solid state (as powder), this cluster is stable in air for approximately 2 weeks, while in solution (in DCM), the cluster is stable for ∼24 h (Figure S10). The partial degradation product of the cluster that forms over time in solution seems to be a result of dissolution because we found no evidence of new cluster types forming (i.e., [Ag_9_(1,2-BDT)_6_](TOA)_3_ was the only cluster species observed).

## Conclusion

In conclusion, we developed an approach to synthesize few-atom (*n* < 10) stable Ag NCs. [Ag_9_(1,2-BDT)_6_]^3–^ is the smallest crystallized Ag NC, with just a single metallically bonded Ag atom at its center. The specific distribution of bidentate thiol ligands on the surface tailors the cluster skeleton into an elongated body-centered cage. The crystal structure shows that the metal core is a combination of two square pyramids that share one vertex capped by six small-footprint dithiolate ligands. Theoretical calculations show that the simulated optical transitions are in good agreement with the experimental optical absorption spectra. These findings pave the way for the development of atomically precise metal NCs with single-digit metallic cores and stimulate research into their catalytic applications.

## Experimental Section

### Chemicals

All chemicals, including silver nitrate (AgNO_3_, 99%), 1,2-benzenedithiol (1,2-BDT), sodium borohydride (NaBH_4_, 99.99% metals basis), and tetraoctylammonium bromide (TOAB), were purchased from Sigma-Aldrich and used without further purification. Solvents, including methanol, dichloromethane (DCM), dimethylformamide (DMF), and acetonitrile, were used from Sigma as received. Distilled water (H_2_O) was obtained from Milli-Q (Millipore apparatus).

### Synthesis and Crystallization of the [Ag_9_(1,2-BDT)_6_](TOA)_3_ NC

This NC was prepared by dissolving AgNO_3_ (20 mg, 0.117 mmol) in methanol. Then, a solution of the 1,2-BDT (13.5 μL, 0.117 mmol) ligand in 10 mL of DCM was added to form a yellow turbid complex, indicating formation of the Ag–S bonds. The reaction mixture was reduced using a fresh aqueous solution of NaBH_4_ (20 mg, 0.5 mmol) in the presence of TOAB (0.4115 mg), resulting in a dark-brown solution that was left under continuous stirring for 90 min at room temperature. To purify the synthesized cluster, we centrifuged the solution at 9500 rpm; the product consisted of a dark-brown precipitate that was neglected, and the dark-brown supernatant was dried under vacuum and washed several times with excess methanol to remove byproducts. The purified NC product (5 mg) was dissolved in DMF (2 mL) and filtered using a syringe filter. The cluster solution was layered with ethanol at 5 °C, forming single crystals (within 1 week) suitable for X-ray crystallography.

## Supporting Information Available

Details of characterization, crystallographic data, and DFT results (PDF)

## Accession Codes

CCDC 2045979 contains the supplementary crystallographic data for this paper. These data can be obtained free of charge via www.ccdc.cam.ac.uk/data_request/cif, or by emailing data_request@ccdc.cam.ac.uk, or by contacting The Cambridge Crystallographic Data Centre, 12 Union Road, Cambridge CB2 1EZ, UK; fax: +44 1223 336033.
