# Supplementary material for: [Ag9(1,2-BDT)6]3–: How Square-Pyramidal Building Blocks Self-Assemble into the Smallest Silver Nanocluster
Source: Inorg Chem. 2021 Mar 17;60(7):4306–12. doi: 10.1021/acs.inorgchem.1c00334 (PMC8041283; doi:10.1021/acs.inorgchem.1c00334)
Supplement: Supplementary file 1 — ic1c00334_si_001.pdf [file ic1c00334_si_001.pdf]

Supporting Information

$[Ag_9(1,2\text{-}BDT)_6]^{3-}$: How Square Pyramidal Building Blocks Self-Assemble into the Smallest Silver Nanocluster

Badriah J. Alamer, [⊥,†,°] *Megalamane S. Bootharaju,* [‡] *Sergey M. Kozlov,* [§] *Zhen Cao,* [⊥,†] *Aleksander Shkurenko,* [⊥,ˆ] *Saidkhodzha Nematulloev,* [⊥,ˆ] *Partha Maity,* [¶,†,⊥] *Omar F. Mohammed,* [¶,†,⊥] *Mohamed Eddaoudi,* [⊥,ˆ] *Luigi Cavallo,* [⊥,†] *Jean-Marie Basset,* [⊥,†] *Osman M. Bakr*[⊥,†,*]

[⊥]Division of Physical Sciences and Engineering, King Abdullah University of Science and Technology (KAUST), Thuwal 23955-6900, Saudi Arabia.

[†]KAUST Catalysis Center (KCC), Division of Physical Sciences and Engineering, King Abdullah University of Science and Technology (KAUST), Thuwal 23955-6900, Saudi Arabia.

[°]Department of Chemistry, College of Sciences, Taif University, Taif, 11099, Saudi Arabia.

[‡]Center for Nanoparticle Research, Institute for Basic Science (IBS), Seoul 08826, Republic of Korea. School of Chemical and Biological Engineering, and Institute of Chemical Processes, Seoul National University, Seoul 08826, Republic of Korea.

[§] Department of Chemical and Biomolecular Engineering, Faculty of Engineering, National University of Singapore, Singapore 119260.

[ˆ] Functional Materials Design, Discovery and Development Research Group (FMD3), Advanced Membranes and Porous Materials Center, King Abdullah University of Science and Technology (KAUST), Thuwal 23955-6900, Saudi Arabia.

[¶]Advanced Membranes and Porous Materials Center (AMPMC), Division of Physical Sciences and Engineering (PSE), King Abdullah University of Science and Technology (KAUST), Thuwal 23955-6900, Saudi Arabia.

Osman M. Bakr: osman.bakr@kaust.edu.sa

Table of Contents

1.0 Materials Characterization……………………………………………………...…S-3

1.1 Ultraviolet-visible Spectroscopy……………………………………….……S-3

1.2 Electrospray Ionization Mass Spectrometry (ESI-MS)………….. …………………S-3

1.3 Single-Crystal X-ray Diffraction Data……………………………………….…S-3

1.4 Photoluminescence spectroscopy..……………………………………………………S-3

1.5 fs-TA spectroscopy……………………………………………………..……….S-4

2.0 Supporting Data………………………………………………………………………S-5

2.1 Synthesis of $[Ag_9(1,2\text{-}BDT)_6](TOA)_3$…………………………………….....…S-5

2.2 UV-vis absorption spectrum………………………………………….……......S-6

2.3 Crystal data and structure refinement…………………………………………….…S-7

2.4 Packing diagram of the unit cell of the cluster…………………………………..…S-8

2.5 Overlay of two crystallographically independent clusters ……………...……….……S-9

2.6 Construction of the metal framework of the cluster …………………………..…S-9

2.7 Photoluminescence (PL) of $[Ag_9(1,2\text{-}BDT)_6](TOA)_3$ NC…………………….…...S-10

2.8 Time-resolved PL of $[Ag_9(1,2\text{-}BDT)_6](TOA)_3$ NC………………...………..........S-11

2.9 Femtosecond transient absorption (fs–TA) spectroscopy………………....….........S-11

2.10. The calculated optical transitions…………………………………………….…..S-12

2.11 Additional frontier molecular orbitals ……………………………………..…..…S-12

2.12 Stability of $[Ag_9(1,2\text{-}BDT)_6](TOA)_3$ NC……………………………...……..…S-13

3.0 Supporting References……………………………………………….……....……S-13

1.0 Materials characterization

1.1 Ultraviolet-visible Spectroscopy

Absorption spectra were recorded with a PerkinElmer Lambda 25 instrument in the spectral range of 200−1100 nm

1.2 Electrospray Ionization Mass Spectrometry (ESI-MS)

ESI mass spectra were recorded using a Bruker MicroTOF-II. The single crystals of nanoclusters were dissolved in a sovent mixture of DMF and acetonitrile (HPLC grade) and the solution was electrosprayed at 300 μL/min flow rate in both positive and negative modes. The instrument parameters were maintained as follows: mass range: 100-10000 Da, capillary voltage: 2.5 kV, nebulizer: 0.1 bar, dry gas: 1.2 L/min at 80-100 ^{o}C.

1.3 Single-crystal X-ray diffraction data

Crystals of $[Ag_9(1,2\text{-BDT})_6](TOA)_3$ was collected on a Bruker X8 PROSPECTOR APEX2 CCD diffractometer at room temperature using Cu *K*α radiation (λ = 1.54178 Å). Indexing was performed using APEX3 (Difference Vectors method).[1] Data integration and reduction were performed using SaintPlus 8.34A.[2] Absorption correction was performed by analytical method implemented in SADABS.[3] Space group was determined using XPREP implemented in APEX2.[1] Structure was solved using SHELXS-97 (direct methods) and refined using SHELXL-2014 (full-matrix least-squares on F^2) contained in WinGX.[4] Crystal data and refinement conditions are shown in Table S1. A full list of restraints and constraints is contained within the CIF file. Due to significant thermal motion and disorder, a set of restraints and constraints was applied to make both geometry and thermal parameters of alkyl chains reasonable (mainly DFIX 1.50(1) and 2.45(2), 1.52(1) and 2.46(2) for 1,2- and 1,3- C-C distances; SIMU 0.02 and RIGU 0.002 for U_{ani}(C)). Occupancies of the disordered parts of the alkyl chains were fixed at 0.5.

1.4 Photoluminescence spectroscopy

Steady state and time resolved PL Spectrometer:

Steady state PL measurement was performed using FluoroMax®-4 spectrometer. Steady state PL measurement a continuous output of 150-W from xenon lamp and single grating excitation and emission monochromators having resolution 0.3 nm, maximum scan seep 80 nms^{-1}, accuracy

±0.5 nm, step size 0.625-100 nm, range 0-950 nm are used inside spectrometer. A calibrated photodiode (R928P) is used to detect the emission photons from the range of 200-850 nm with a linearity of 2 X 10^{16} counts s^{-1} (<100 dark counts s^{-1}). Time resolved PL measurement, the instrument works on the principle of time correlated single photon counting (TCSPC). In the present work, 450 nm laser pulses was used as the excitation light sources and a TBX-04 detection module coupled with a special Hamamatsu PMT was used for photons detection. The PL decays were detected at 790 nm (instrument detection limit). The decay trace was fitted using the exponential equation $I(t) = \sum_i^n \alpha_i \, e^{-t/\tau_i}$ where, *I (t)* is the total intensity remaining at time t. Where, α_i and τ_i are the amplitude and decay time of ith component, respectively. The average lifetime of the measured samples is calculated using $\tau_{avg} = \sum_i^n \frac{\alpha_i \tau_i}{\alpha_i}$ equation.

1.5 fs-TA spectroscopy

The fs-TA spectroscopy were performed on timescales of 0.1 ps to 6 ns, which is based on a multipass amplified Ti:sapphire laser (800 nm laser pulses of 7 mJ/pulse energy of ~100 fs pulse width having 1 kHz repetition rate, Astrella from Coherent), and in conjunction with Helios spectrometers. The excitation pump pulses at 450 were generated after passing through a fraction of 800 nm beam into the spectrally tunable (240−2600 nm) optical parametric amplifier (Newport Spectra-Physics). The pump fluence of the excitation laser source was adjusted by using neutral density (ND) filter to avoid the multiple charge carriers generation. The probe pulses (UV visible and NIR wavelength continuum, white light) were generated by passing another fraction of the 800 nm pulses through the 2-mm thick calcium fluoride (CaF_2) crystal. Before white light generation, the 800 nm amplified pulses were passed through a motorized delay stage. Depending on the movement of delay stage, the transient species were detected following excitation at different time scales. The white light was split into two beams (named as signal and reference) and focused on two fiber optics for the improvement of better signal to noise ratio. The excitation pump pulses were spatially overlapped with the probe pulses on the samples after passing through a synchronized mechanical chopper (500 Hz), which blocked an

alternative pump pulses. The absorption changed (ΔA) was measured with respect to the time delay and wavelength (λ). All spectra were averaged over a time period of 2 s for each time delay.

Note: It is advised to use to wear appropriate eye-protection during the laser experiments.

2.0 Supporting Data

2.1 Synthesis of $[Ag_9(1,2\text{-BDT})_6](TOA)_3$.

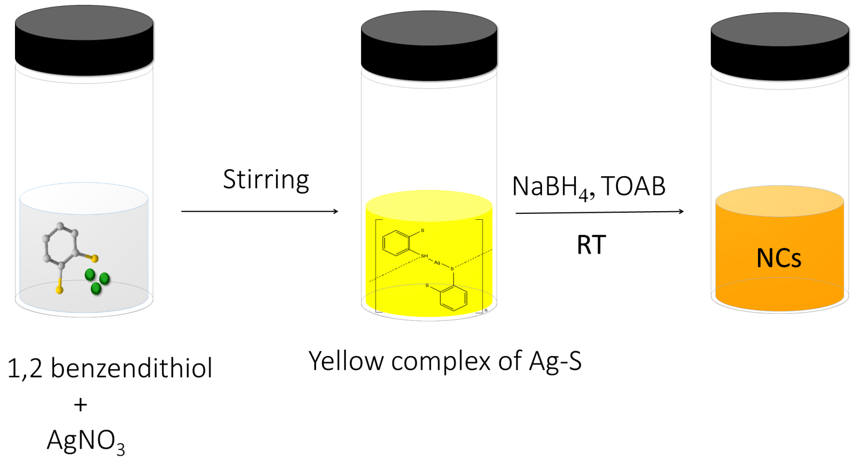

Figure S1. Synthesis of $[Ag_9(1,2\text{-BDT})_6](TOA)_3$ nanoclusters (NCs).

2.2 UV-vis absorption spectrum of $[Ag_9(1,2\text{-}BDT)_6](TOA)_3$.

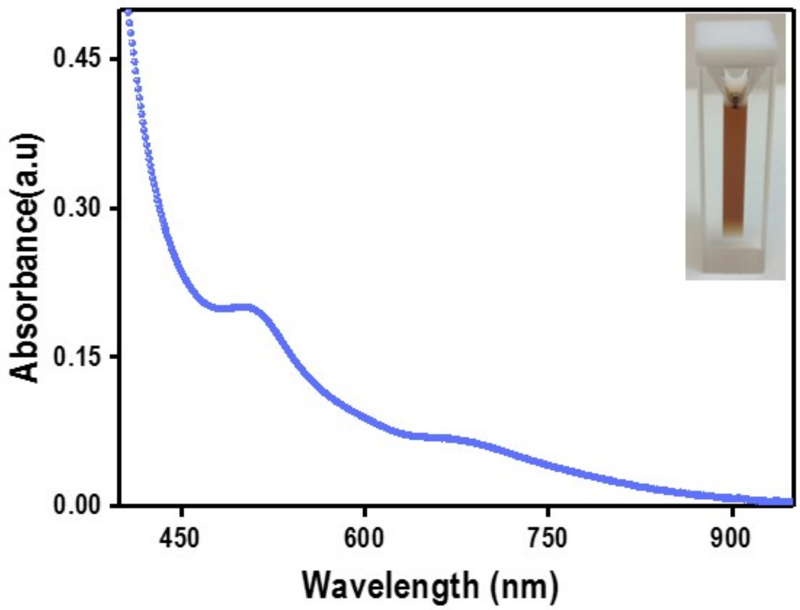

Figure S2. UV-vis absorption spectrum of $[Ag_9(1,2\text{-}BDT)_6](TOA)_3$ cluster. Inset: a photograph showing actual color of the synthesized cluster in DCM.

2.3 Crystal data and structure refinement.

Table S1. Crystal data and structure refinement conditions for $[Ag_9(1,2\text{-BDT})_6](TOA)_3$.

Empirical formula	$C_{132}H_{228}Ag_9N_3S_{12}$
Formula weight	3212.71
Crystal system, space group	Monoclinic, $P2_1/c$
Unit cell dimensions	a = 29.294(1) Å, b = 26.239(1) Å, c = 18.8937(8) Å, β = 96.308(2)°
Volume	14435(1) Å^3
Z, calculated density	4, 1.478 Mg m^{-3}
F(000)	6624
Temperature (K)	296(2)
Radiation type, λ	Cu $K\alpha$,1.54178 Å
Absorption coefficient	11.53 mm^{-1}
Absorption correction	Multi-scan
Max and min transmission	0.149 and 0.036
Crystal size	0.20 × 0.25 × 0.30 mm
Shape, colour	Prism, yellow
θ range for data collection	2.3–66.7°
Limiting indices	$-29 \le h \le 30$, $-16 \le k \le 27$, $-19 \le l \le 19$
Reflection collected / unique / observed with $I > 2\sigma(I)$	147349 / 17034 (R_{int} = 0.068) / 12840
Completeness to Θ_{full} = 53.4°	99.7 %
Refinement method	Full-matrix least-squares on F^2
Data / restraints / parameters	17034 / 1392 / 1579
Final R indices [$I > 2s(I)$]	R_1 = 0.072, wR_2 = 0.206
Final R indices (all data)	R_1 = 0.092, wR_2 = 0.218
Weighting *scheme*	$[\sigma^2(F_o^2) + (0.1037P)^2 + 69.0115P]^{-1*}$
Goodness-of-fit	1.05
Largest diff. peak and hole	0.84 and -0.57 e Å^{-3}

$^*P = (F_o^2 + 2F_c^2)/3$

2.4 Packing diagram of the unit cell of the cluster.

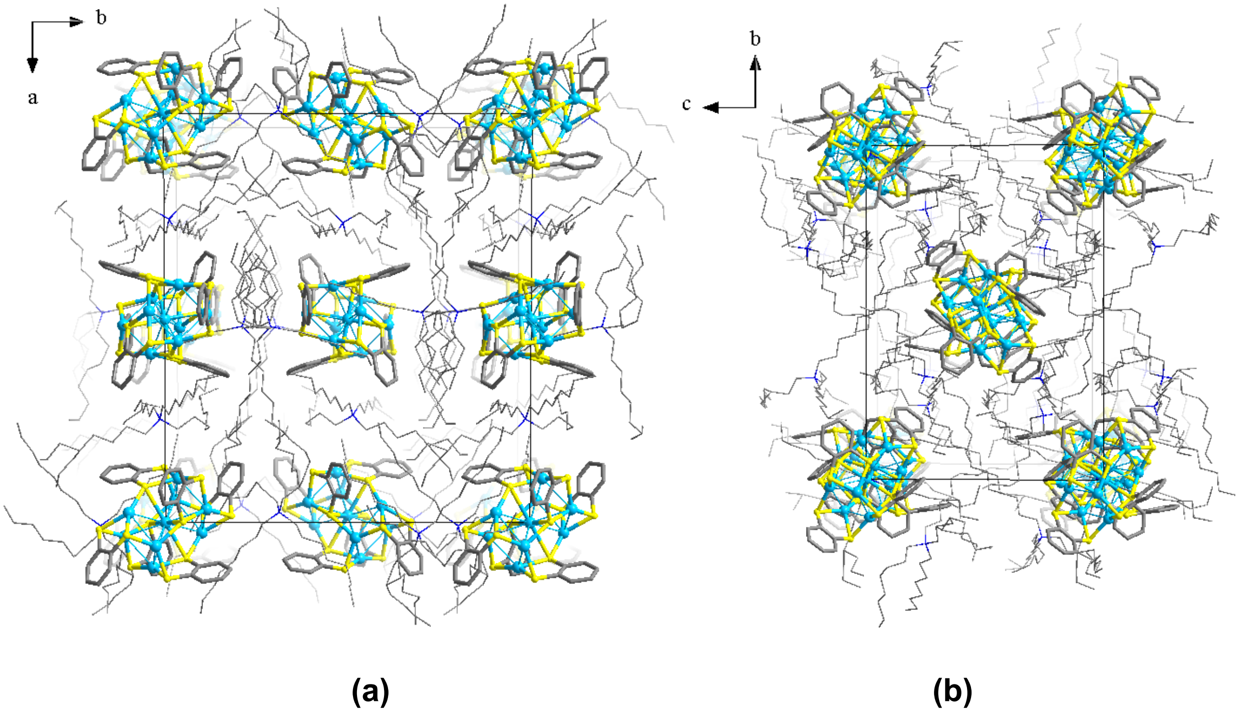

Figure S3. Packing diagram of $[Ag_9(1,2\text{-}BDT)_6](TOA)_3$ cluster: (a) view along the *c* axis; (b) view along the *a* axis. H-atoms are omitted for clarity.

2.5 Overlay of two crystallographically independent clusters.

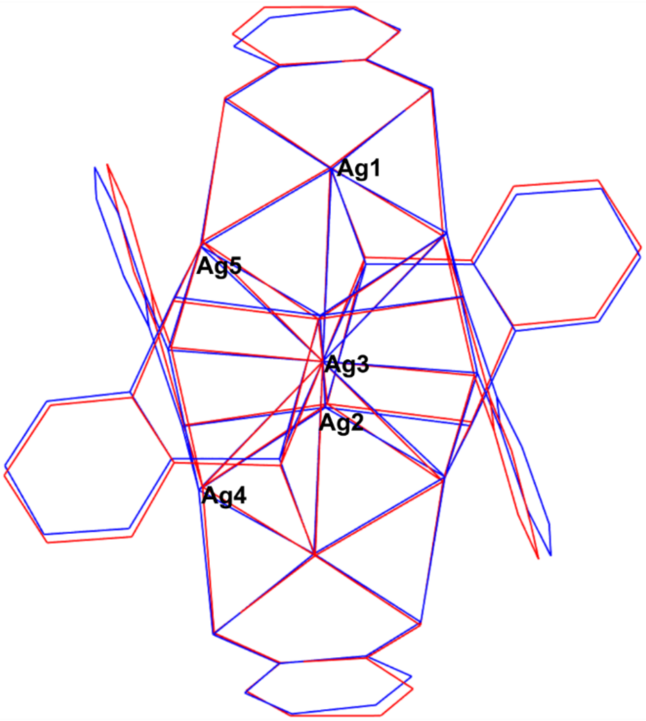

Figure S4. Overlay of two crystallographically independent $[Ag_9(1,2\text{-BDT})_6]^{3-}$ clusters. The cluster I is in red and the cluster II is in blue. H-atoms are omitted for clarity.

2.6 Construction of the metal framework of the cluster.

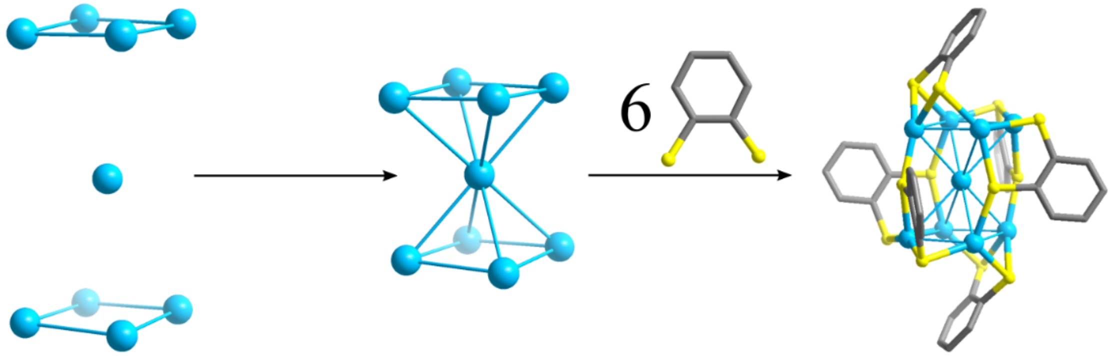

Figure S5. Construction of the metal framework of $[Ag_9(1,2\text{-BDT})_6](TOA)_3$ cluster. H-atoms are omitted for clarity.

2.7 Photoluminescence (PL) of $[Ag_9(1,2\text{-}BDT)_6](TOA)_3$ NC.

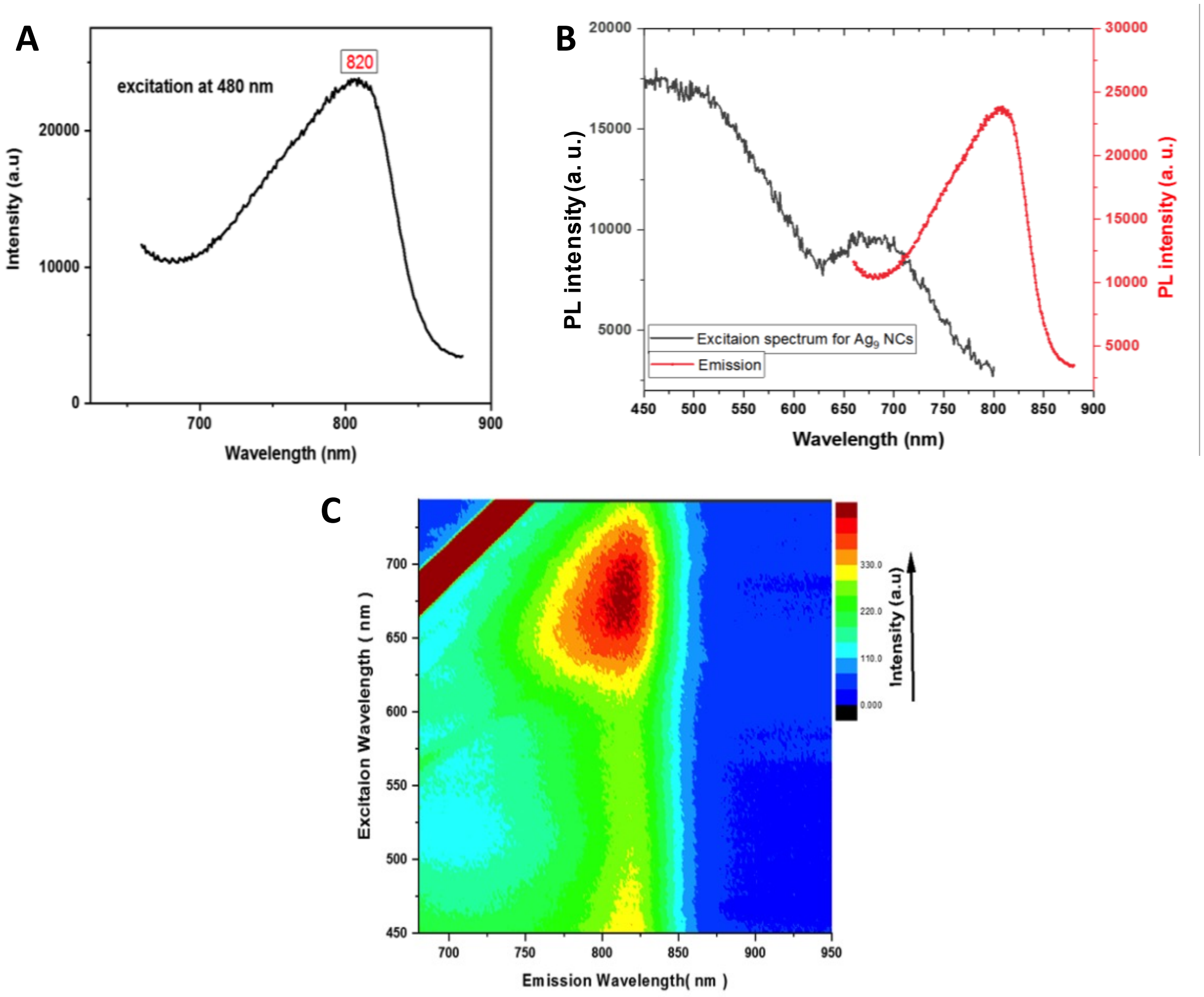

Figure S6. (A) PL emission (480 nm excitation), (B) PL excitation (820 nm emission) and PL emission (480 nm excitation) spectra of $[Ag_9(1,2\text{-}BDT)_6](TOA)_3$ cluster. (C) 3D- excitation-emission matrix of $[Ag_9(1,2\text{-}BDT)_6](TOA)_3$ NC at 293 K.

2.8 Time-resolved PL of $[Ag_9(1,2\text{-}BDT)_6](TOA)_3$ NC.

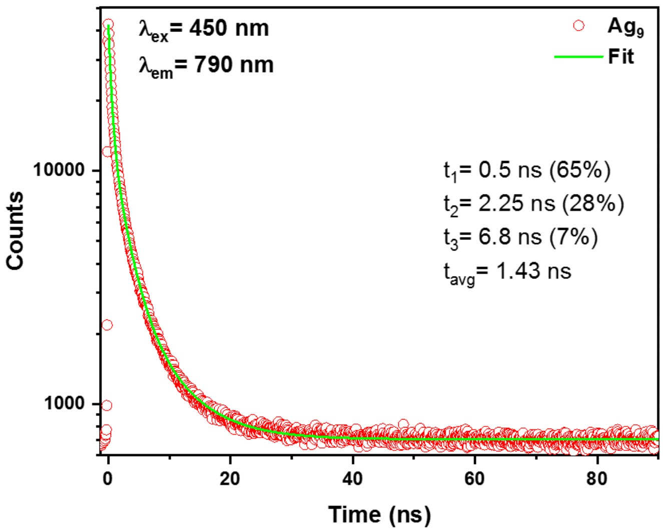

Figure S7. The PL decay trace of Ag_9 NC in DCM monitored at 790 nm after 450 nm laser excitation at room temperature.

2.9 Femtosecond transient absorption (fs–TA) spectroscopy.

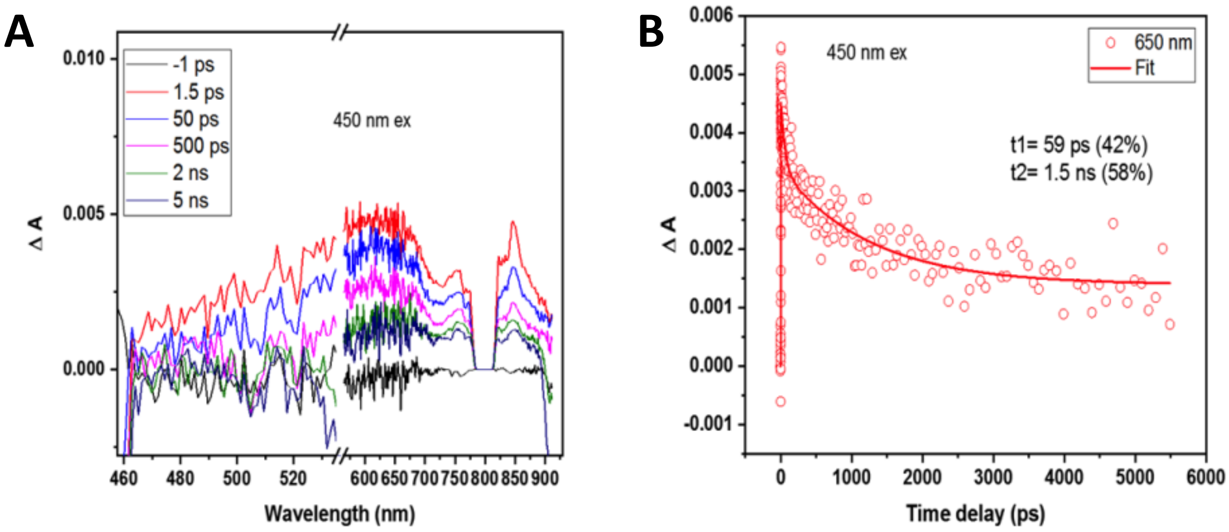

Figure S8. (A) Time-evolution fs-TA spectra of Ag_9 NC. (B) Kinetic traces probed at 650 nm in response to 450 nm optical excitation. The solid line in B represents the exponential fit of the experimental data.

2.10 The calculated optical transitions.

Table S2. The calculated optical transitions with the highest contributions to the optical absorption features.

Experimental Feature	Transition (eV)	Oscillator Strength (dimensionless)	Molecular Orbitals with Contributions above 10%
Lowest E shoulder	1.86	0.017	HOMO → LUMO (99%)
Low E shoulder	2.38	0.082	HOMO-6 → LUMO (69%)<br>HOMO-3 → LUMO+1 (15%)<br>HOMO-4 → LUMO+1 (10%)
Unobserved	2.70	0.13669	HOMO-7 → LUMO+1 (86%)
Main peak	3.15	0.12951	HOMO-12 → LUMO+1 (87%)

2.11 Additional frontier molecular orbitals.

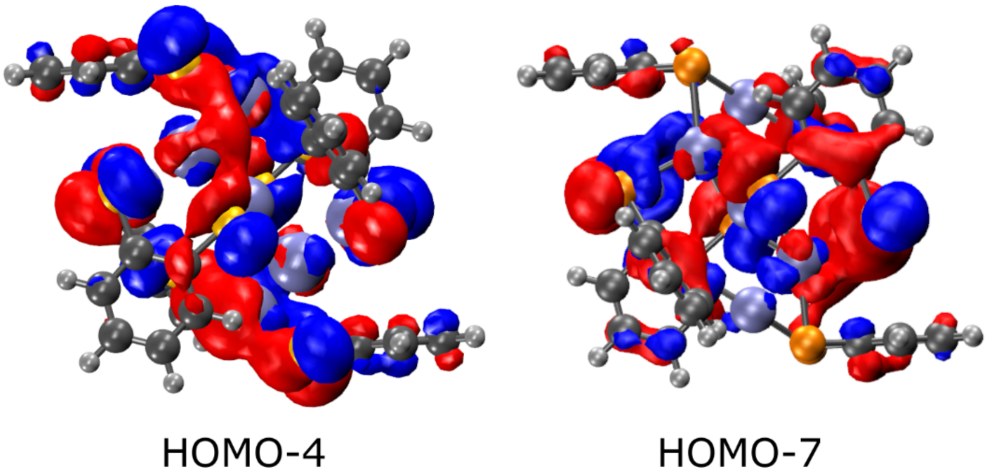

Figure S9. Additional molecular orbitals involved in the prominent optical transitions.

2.12 Stability of $[Ag_9(1,2\text{-}BDT)_6](TOA)_3$ NC.

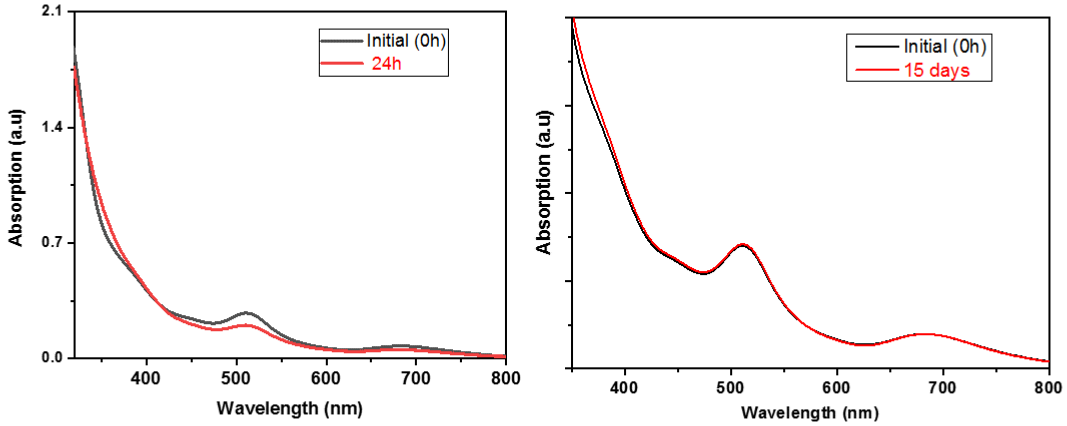

Figure S10. The time evolution of the UV-Vis absorption spectrum of $[Ag_9(1,2\text{-}BDT)_6](TOA)_3$ NC after storage in (A) solution-state in DCM and (B) solid-state. The spectra in B were measured by redissolving the cluster powder in DCM 15 days after synthesis.

3.0 Supporting References

1. APEX3. Bruker AXS Inc, Madison, Wisconsin, USA, 2017.
2. SAINT. Bruker AXS. Inc, Madison, Wisconsin, USA, 2014.
3. SADABS. G. M. Sheldrick. University of Gottingen, Germany, 2012.
4. SHELXS-97, SHELXL-2014/7. G. M. Sheldrick, *Acta Cryst.* 2015, C**71**, 3-8; WinGX. L. J. Farrugia, *J. Appl. Cryst.* 2012, **45**, 849-854.
